# Reclassification of the Fuhrman grading system in renal cell carcinoma-does it make a difference?

**DOI:** 10.1186/2193-1801-2-378

**Published:** 2013-08-10

**Authors:** Tahir Qayyum, Peter McArdle, Clare Orange, Morag Seywright, Paul Horgan, Grenville Oades, Michael Aitchison, Joanne Edwards

**Affiliations:** Unit of Experimental Therapeutics, Institute of Cancer, College of MVLS, University of Glasgow, Western Infirmary, Glasgow, G11 6NT Scotland, UK; Department of Urology, Gartnaval General Hospital, Glasgow, Scotland, UK; Department of Pathology, Western Infirmary, Glasgow, Scotland, UK; School of Medicine, College of MVLS, University of Glasgow, Royal Infirmary, Glasgow, Scotland, UK

**Keywords:** Renal cell cancer, Fuhrman grade

## Abstract

**Purpose:**

The aim of this study was to determine whether reclassifying the Fuhrman grading system provides further prognostic information.

**Materials and methods:**

We studied the pathological features and cancer specific survival of 237 patients with clear cell cancer undergoing surgery between 1997–2007 in a single centre. The original Fuhrman grading system was investigated as well as various simplified models utilising the original Fuhrman grade.

**Results:**

The median follow up was 69 months. On univariate analysis, the conventional Fuhrman grading system as well various simplified models were predicative of cancer specific survival. On multivariate analysis, only the three tiered modified model in which grades 1 and 2 were combined whilst grades 3 and 4 were kept separate was an independent predictor of cancer specific survival (p=0.001, HR 2.17, 95% CI 1.37-3.43). Furthermore this simplified model demonstrated a stronger relationship to recurrence than the conventional 4 tiered Fuhrman grading system.

**Conclusions:**

A modified, three-tiered Fuhrman grading system has been demonstrated to be an independent predictor of cancer specific survival.

## Introduction

In the UK alone, approximately 9000 new cases of renal cancer are diagnosed each year and nearly 4000 die of their disease (www.cancerresearchuk.org). Overall survival is poor, even for those patients who undergo resection; the estimated 5 year survival rate is only 50%.

Currently, the TNM stage and tumour grade are the most widely used tools to predict survival. Various grading classifications for clear cell carcinoma based on morphological features have been proposed (Arner et al. 1965; Delahunt & Nacey 1987; Fuhrman et al. 1982; Lohse et al. 2002; Skinner et al. 1971; Syrjanen & Hjelt 1978; Thoenes et al. 1986) and of these the Fuhrman grading system (Fuhrman et al. 1982) has achieved widespread usage in pathology practise. The Fuhrman grading system has been demonstrated to be an independent predictor of survival (Ficarra et al. 2001) having been acknowledged as optimal for predicting outcome (Bostwick & Murphy 1998) and therefore has been incorporated into the majority of prognostic algorithms including Ssign (Frank et al. 2002), UISS (Zisman et al. 2001) and Leibovich (Leibovich et al. 2003).

The Fuhrman grading system is based on assessment of the uniformity of nuclear size, nuclear shape and nucleolar prominence (Fuhrman et al. 1982). The Fuhrman grading system has been demonstrated to correlate to metastasis with grade 1 tumours having a statistically significant lower metastases rate compared to those with grade 2 to 4 and survival rates being distinguished into 3 categories, those with grade 1, those with grade 4 and those with grades 2 and 3 (Fuhrman et al. 1982). Despite the popularity of this grading system, problems have been demonstrated regarding its application (Delahunt & Nacey 1987; Delahunt 1998; Medeiros et al. 1997).

There has been suggestions that the Fuhrman grading system has low-moderate inter-observer agreement (Medeiros et al. 1997; Lang et al. 2005; Al-Aynati et al. 2003; Bektas et al. 2009) and that a simplified system improves inter-observer agreement (Lang et al. 2005; Al-Aynati et al. 2003) as well as demonstrating as much accuracy as the conventional grading system (Sun et al. 2009; Rioux-Leclercq et al. 2007). Furthermore there are those suggesting that the ideal grading system is yet to be defined and should consist of three tiers (Medeiros et al. 1997) whilst a three tired system has been shown to be an independent predictor of survival (Ficarra et al. 2005; Hong et al. 2011). Given the evidence suggesting that a simplified system improves the prognostic ability of the Fuhrman grading, we aim to evaluate which if any simplified system would further aid in determining prognosis.

## Materials and methods

Patients with clear cell renal cancer were included for this study. These patients had undergone resection based on the surgical findings and the results of CT scans for staging purposes between January 1997 and Dec 2007 in the North Glasgow NHS Trust. The Research Ethics Committee of West of Scotland has approved the study.

Two hundred and thirty seven patients with clear cell renal cancer were identified retrospectively that underwent nephrectomy. The study cohort constituted a representative sample of all surgically treated patients within this period.

Clinicopathological data including T stage, nuclear grade assessment (Fuhrman et al. 1982) and survival for each patient was collected. Survival was determined from the time of surgical treatment to the time of last follow up. The cause of death was determined by linkage through the Scottish Cancer Registry. In those who were deceased, if the primary cause of death was of renal cancer, these were classed as cancer specific and all other causes were non-cancer specific deaths. Patients notes were accessed for documented evidence of recurrence otherwise they were deemed to have no recurrences.

The original Fuhrman grading system was investigated as well as various simplified systems utilising the Fuhrman grade. Table 1 shows the various simplified models that were investigated.

**Table 1:** **Demonstrating the various simplified grading systems investigated**

Fuhrman grading system	1	2	3	4
Grading system 1	1		2	
Grading system 2	1	2		
Grading system 3	1			2
Grading system 4	1	2	3	
Grading system 5	1		2	3

Statistical analysis was undertaken using SPSS (Chicago, IL, USA). Cancer specific survival rates were generated using the Kaplan Meir method. The log rank test was utilised to compare significant differences between subset groups using univariate analysis. Multivariate analysis was carried out based on the results of the univariate analysis. Multivariate Cox regression analysis was performed to identify those factors that were independently associated with cancer specific death. A stepwise backward procedure was utilised to ascertain which of the variables had a significant independent relationship with survival.

## Results

The patient characteristics are shown in Table 2. The median follow up was 69 (2.1-181) months. The median age was 60 (23–86) years. Thirty three patients died of their disease. Within the cohort, the most common tumour stage was pT1 (47%). The most common Fuhrman grades were II (36%) and III (41%). Pathology slides were reviewed by a single Uro-Pathologist.

**Table 2:** **Relationship between clinicopathological characteristics and cancer specific survival**

Variable	Numbers	
		p-value
Age (<60/>60)	108/129	0.918
T Stage (1/2/3/4)	112/35/85/5	**<0.001**
Nuclear grade (1/2/3/4)	25/86/97/29	**0.005**
Recurrence (No/Yes)	178/59	**<0.001**

**Figure 1:**
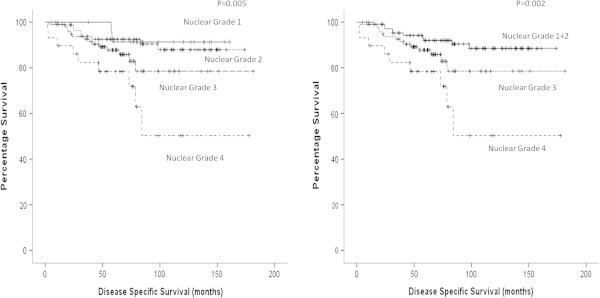
**Kaplan Meier graphs demonstrating the conventional 4 tiered Fuhrman grading system (p=0.005) and a simplified model where grades 1 and 2 are combined and grades 3 and 4 are kept separate (p=0.002) against disease specific survival.**

Univariate analysis of potential predictors of cancer specific survival showed that the majority of the grading models were statistically significant predictors of cancer specific survival (Table 3, Figure 1). On multivariate analysis of those that were significant on univariate, only model 5 which is a modified three tired model combining grades 1 and 2 whilst grades 3 and 4 are kept as separate was found to be an independent prognostic factor in its association with cancer specific survival (p=0.001, HR 2.17, 95% CI 1.37-3.43, Table 3).

**Table 3:** **Relationship between various simplified nuclear grading systems and cancer specific survival**

Variable	Numbers	Univariate analysis	Multivariate analysis	
		p-value	p-value	HR
Nuclear grade (1/2/3/4)	25/86/97/29	**0.005**		
Grading system 1 ((1+2)/(3+4))	111/126	**0.008**		
Grading system 2 ((1/(2+3+4))	25/212	0.237		
Grading system 3 ((1+2+3)/4)	208/29	**0.002**		
Grading system 4 (1/2/(3+4))	25/86/126	**0.029**		
Grading system 5 ((1+2)/3/4)	111/97/29	**0.002**	**0.001**	2.17 (1.37-3.43)

On *x*^2^ analysis of the various simplified grading models, whilst majority of the grading models demonstrated a positive correlation with T Stage (Table 4), model 3, which is a two tired model combining grades 1, 2 and 3 whilst grade 4 is kept separate demonstrated the strongest correlation to T Stage (p<0.001, Table 4). When analysing the grading models, the majority of these demonstrated a positive correlation to recurrence, whilst models 3 and 5 demonstrated the strongest correlation to this clinicopathological factor (p<0.001, Table 4).

**Table 4:** **Interrelationship between clinicopathological characteristics of patients and various simplified nuclear grading systems**

Variable	Numbers	T stage	Recurrence
Nuclear grade (1/2/3/4)	25/86/97/29	**0.001**	**0.001**
Grading system 1 ((1+2)/(3+4))	111/126	**0.029**	**0.01**
Grading system 2 ((1/(2+3+4))	25/212	0.07	0.278
Grading system 3 ((1+2+3)/4)	208/29	**<0.001**	**<0.001**
Grading system 4 (1/2/(3+4))	25/86/126	**0.015**	**0.016**
Grading system 5 ((1+2)/3/4)	111/97/29	**0.001**	**<0.001**

## Discussion

In this cohort of patients with clear cell carcinoma, a simplified 3-tiered model where grades 1 and 2 are combined whilst grades 3 and 4 were kept separate (model 5) was an independent predictor of cancer specific survival on multivariate analysis. Furthermore, this modified model was also one of only two to correlate to disease recurrence.

Several studies have demonstrated that the Fuhrman grading system is capable of predicting cancer specific survival independent of pathological stage (Frank et al. 2002; Patard et al. 2003). Studies however have demonstrated that the conventional Fuhrman grading system is complex (Ficarra et al. 2005; Medeiros et al. 1988) and that a simplified system improves inter-observer agreement (Lang et al. 2005; Al-Aynati et al. 2003). It has previously been demonstrated that combining grades 1 and 2 improves the prognostic ability of the Fuhrman grading system and a three tiered system combining grades 1 and 2 whilst keeping grades 3 and 4 separate is an independent predictor of survival (Ficarra et al. 2005; Hong et al. 2011), a finding similar to that reported in this study. There has been reports that this simplified three tiered model has a similar performance in multivariate models predicting outcome to the conventional 4 tiered Fuhrman system (Sun et al. 2009; Rioux-Leclercq et al. 2007). In terms of cancer specific survival, the gap between grades 3 and 4 was more evident than the gap between grades 1 and 2. This result is similar to that demonstrated by several other studies (Sun et al. 2009; Rioux-Leclercq et al. 2007; Ficarra et al. 2005; Hong et al. 2011) where a three tiered model was proposed (Ficarra et al. 2005; Hong et al. 2011). This further shows that grades 3 and 4 would be less suitable for combining than grades 1 and 2 and strengthens the argument for a three tiered model.

As with most studies examining the modification of the Fuhrman grading system, the present study is limited due its retrospective nature with no analysis of inter or intra observer variability when assigning the Fuhrman grade and no external review of nuclear grade.

In the present study, a simplified version of the Fuhrman grading system whereby grades 1 and 2 are combined and grades 3 and 4 were kept separate was shown to be an independent predictor of cancer specific survival and demonstrated a positive correlation to disease recurrence suggesting that this modified model can be considered an option for the purposes of prognosis in those with clear cell renal cancer. Further work is required in terms of a prospective study for validation.

## References

[CR1] Al-Aynati M, Chen V, Salama S, Shuhaibar H, Treleaven D, Vincic L (2003). Interobserver and intraobserver variability using the Fuhrman grading system for renal cell carcinoma. Arch Pathol Lab Med.

[CR2] Arner O, Blanck C, Von ST (1965). Renal adenocarcinoma; morphology–grading of malignancy–prognosis. A study of 197 cases. Acta Chir Scand Suppl.

[CR3] Bektas S, Bahadir B, Kandemir NO, Barut F, Gul AE, Ozdamar SO (2009). Intraobserver and interobserver variability of Fuhrman and modified Fuhrman grading systems for conventional renal cell carcinoma. Kaohsiung J Med Sci.

[CR4] Bostwick DG, Murphy GP (1998). Diagnosis and prognosis of renal cell carcinoma: highlights from an international consensus workshop. Semin Urol Oncol.

[CR5] Delahunt B (1998). Histopathologic prognostic indicators for renal cell carcinoma. Semin Diagn Pathol.

[CR6] Delahunt B, Nacey JN (1987). Renal cell carcinoma. II. Histological indicators of prognosis. Pathology.

[CR7] Ficarra V, Righetti R, Martignoni G, D'Amico A, Pilloni S, Rubilotta E, Malossini G, Mobilio G (2001). Prognostic value of renal cell carcinoma nuclear grading: multivariate analysis of 333 cases. Urol Int.

[CR8] Ficarra V, Martignoni G, Maffei N, Brunelli M, Novara G, Zanolla L, Pea M, Artibani W (2005). Original and reviewed nuclear grading according to the Fuhrman system: a multivariate analysis of 388 patients with conventional renal cell carcinoma. Cancer.

[CR9] Frank I, Blute ML, Cheville JC, Lohse CM, Weaver AL, Zincke H (2002). An outcome prediction model for patients with clear cell renal cell carcinoma treated with radical nephrectomy based on tumor stage, size, grade and necrosis: the SSIGN score. J Urol.

[CR10] Fuhrman SA, Lasky LC, Limas C (1982). Prognostic significance of morphologic parameters in renal cell carcinoma. Am J Surg Pathol.

[CR11] Hong SK, Jeong CW, Park JH, Kim HS, Kwak C, Choe G, Kim HH, Lee SE (2011). Application of simplified Fuhrman grading system in clear-cell renal cell carcinoma. BJU Int.

[CR12] Lang H, Lindner V, de Fromont Molinie V, Letourneux H, Meyer N, Martin M, Jacqmin D (2005). Multicenter determination of optimal interobserver agreement using the Fuhrman grading system for renal cell carcinoma: Assessment of 241 patients with > 15-year follow-up. Cancer.

[CR13] Leibovich BC, Blute ML, Cheville JC, Lohse CM, Frank I, Kwon ED, Weaver AL, Parker AS, Zincke H (2003). Prediction of progression after radical nephrectomy for patients with clear cell renal cell carcinoma: a stratification tool for prospective clinical trials. Cancer.

[CR14] Lohse CM, Blute ML, Zincke H, Weaver AL, Cheville JC (2002). Comparison of standardized and nonstandardized nuclear grade of renal cell carcinoma to predict outcome among 2,042 patients. Am J Clin Pathol.

[CR15] Medeiros LJ, Gelb AB, Weiss LM (1988). Renal cell carcinoma. Prognostic significance of morphologic parameters in 121 cases. Cancer.

[CR16] Medeiros LJ, Jones EC, Aizawa S, Aldape HC, Cheville JC, Goldstein NS, Lubensky IA, Ro J, Shanks J, Pacelli A, Jung SH (1997). Grading of renal cell carcinoma: Workgroup No. 2. Union Internationale Contre le Cancer and the American Joint Committee on Cancer (AJCC). Cancer.

[CR17] Patard JJ, Leray E, Rodriguez A, Rioux-Leclercq N, Guille F, Lobel B (2003). Correlation between symptom graduation, tumor characteristics and survival in renal cell carcinoma. Eur Urol.

[CR18] Rioux-Leclercq N, Karakiewicz PI, Trinh QD, Ficarra V, Cindolo L, de la Taille A, Tostain J, Zigeuner R, Mejean A, Patard JJ (2007). Prognostic ability of simplified nuclear grading of renal cell carcinoma. Cancer.

[CR19] Skinner DG, Colvin RB, Vermillion CD, Pfister RC, Leadbetter WF (1971). Diagnosis and management of renal cell carcinoma. A clinical and pathologic study of 309 cases. Cancer.

[CR20] Sun M, Lughezzani G, Jeldres C, Isbarn H, Shariat SF, Arjane P, Widmer H, Pharand D, Latour M, Perrotte P, Patard JJ, Karakiewicz PI (2009). A proposal for reclassification of the Fuhrman grading system in patients with clear cell renal cell carcinoma. Eur Urol.

[CR21] Syrjanen K, Hjelt L (1978). Grading of human renal adenocarcinoma. Scand J Urol Nephrol.

[CR22] Thoenes W, Storkel S, Rumpelt HJ (1986). Histopathology and classification of renal cell tumors (adenomas, oncocytomas and carcinomas). The basic cytological and histopathological elements and their use for diagnostics. Pathol Res Pract.

[CR23] http://www.cancerresearchuk.orghttp://www.cancerresearchuk.org

[CR24] Zisman A, Pantuck AJ, Dorey F, Said JW, Shvarts O, Quintana D, Gitlitz BJ, Dekernion JB, Figlin RA, Belldegrun AS (2001). Improved prognostication of renal cell carcinoma using an integrated staging system. J Clin Oncol.

